# Modeling epigenome folding: formation and dynamics of topologically associated chromatin domains

**DOI:** 10.1093/nar/gku698

**Published:** 2014-08-04

**Authors:** Daniel Jost, Pascal Carrivain, Giacomo Cavalli, Cédric Vaillant

**Affiliations:** 1 Laboratoire de Physique, Ecole Normale Supérieure de Lyon, CNRS UMR 5672, Lyon 69007, France; 2 Institute of Human Genetics, CNRS UPR 1142, Montpellier 34000, France

## Abstract

Genomes of eukaryotes are partitioned into domains of functionally distinct chromatin states. These domains are stably inherited across many cell generations and can be remodeled in response to developmental and external cues, hence contributing to the robustness and plasticity of expression patterns and cell phenotypes. Remarkably, recent studies indicate that these 1D epigenomic domains tend to fold into 3D topologically associated domains forming specialized nuclear chromatin compartments. However, the general mechanisms behind such compartmentalization including the contribution of epigenetic regulation remain unclear. Here, we address the question of the coupling between chromatin folding and epigenome. Using polymer physics, we analyze the properties of a block copolymer model that accounts for local epigenomic information. Considering copolymers build from the epigenomic landscape of *Drosophila*, we observe a very good agreement with the folding patterns observed in chromosome conformation capture experiments. Moreover, this model provides a physical basis for the existence of multistability in epigenome folding at sub-chromosomal scale. We show how experiments are fully consistent with multistable conformations where topologically associated domains of the same epigenomic state interact dynamically with each other. Our approach provides a general framework to improve our understanding of chromatin folding during cell cycle and differentiation and its relation to epigenetics.

## INTRODUCTION

Gene expression is regulated by many sets of proteins that associate with the genome in a cell-type and condition-specific manner at specific regulatory elements including proximal promoters, enhancers and repressors. The packaging of eukaryotic DNA into chromatin contributes to this regulation via the modulation of the accessibility and specificity of regulators to their nucleic sites. Locally, the chromatin state is characterized by various features like the nucleosome positioning, the covalent modifications of DNA and histones tails and the insertion of histone variants. This pattern of chromatin states along the genome, the so-called ‘epigenome’, is itself regulated by the combined action of different specialized chromatin regulators like chromatin remodelers, modifying enzymes and histone chaperones.

The general picture that emerges from the genome-wide high-resolution profiling of structural and functional chromatin marks obtained in various organisms and cell types (1–4), is that eukaryotic genomes are linearly organized into distinct epigenomic domains. These domains extend over few kilobases up to few megabases, are characterized by a specific type of chromatin and are isolated from their neighborhood by boundary elements such as insulators. Euchromatin, less condensed, early replicating and containing most active genes, is generally distinguished from heterochromatin, typically highly condensed, late replicating and inhibitory to transcriptional machinery. In many higher eukaryotes, from plants to mammals, statistical analyses of hundreds of chromatin marks have identified only a small number of main chromatin types (1,3,5,6), typically four or five, covering the well-known constitutive HP1-like heterochromatin or the facultative (developmentally regulated) Polycomb-like heterochromatin but also a less-characterized ultra-repressive heterochromatin enriched in genes that are expressed in very few tissues, the so-called void or black chromatin (1,7).

Interestingly, within epigenomic domains, regulatory sequences such as enhancers may be located far from the target genes and multiple elements that are distributed over large regions may collaborate or compete for the regulation of individual genes or gene clusters. This implies the existence of long-range mechanisms where regulatory elements could act over large genomic distances up to hundreds of kilobases or more. A possible mechanism regulating such long-range effects is the linear spreading of a regulatory signal (e.g. repressive chromatin state) from nucleation sites (e.g. silencers) to target-sites (e.g. promoters). Another non-exclusive mechanism calls into play the polymeric nature of chromatin that may induce spatial colocalization of regulatory sequences with their target. Recently, chromosome conformation capture (3C)-based studies have indeed shown that regulatory elements can act over large genomic distances by chromatin looping (8,9) forming active or repressive higher-order chromatin structure at particular developmentally regulated genes. These pairwise 3D interactions are mediated by DNA binding proteins such as insulators or cohesin and mediator (10) that would cluster in space and bridge distant regulatory sites. At a genomic scale, the contact maps of *Drosophila* (11,12), mouse (13) and human (13,14) chromosomes have further revealed a remarkable 3D compartmentalization where epigenomic domains fold into independent ‘spatial domains’, the so-called topologically associated domains (TADs), characterized by (i) high intra-domain contact frequencies; (ii) 3D insulation between adjacent domains; (iii) and in many cases, significant contacts between distal domains of the same chromatin type (Figures 3A and 4A). This compartmentalization is consistent with the nuclear structure, as revealed by imaging techniques such as electron microscopy and immuno-FISH (15–17), that clearly shows a phase separation between euchromatin versus heterochromatin and to some extent between the different heterochromatin types (18).

**Figure 1.**
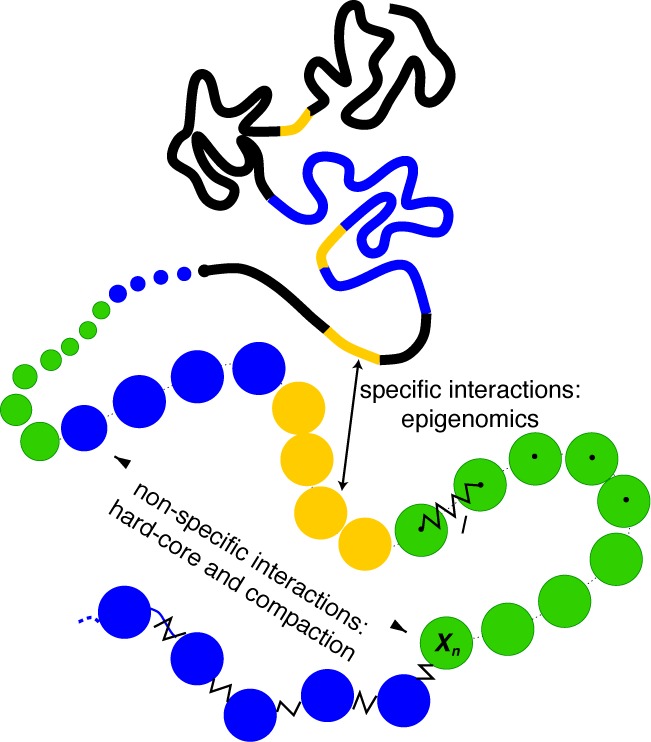
Block copolymer model: the chromatin is modeled as a self-avoiding bead-spring chain where each monomer represents a portion of DNA (10 kb) and is characterized by its epigenetic state: yellow (active), green (HP1-like heterochromatin), blue (Polycomb-like heterochromatin), black (repressive chromatin) (1). The model integrates non-specific and specific short-range interactions to account respectively for the effective compaction of the chain and for epigenomically related affinities between monomers.

**Figure 2.**
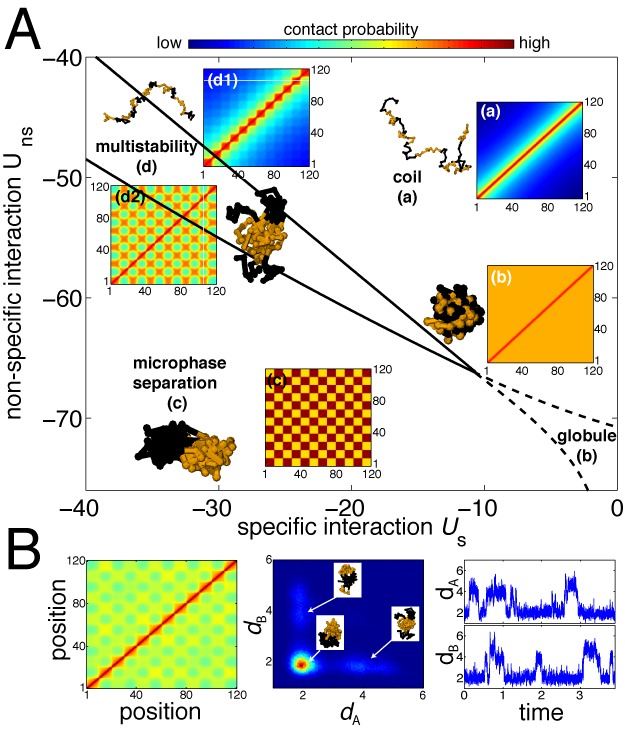
(**A**) Phase diagram of the copolymer (A_10_B_10_)_6_ as a function of the strength of specific and non-specific interactions (in *k*_B_*T* unit). Insets represent typical heat maps of the probability of contacts between two monomers (in log-unit) for the different phases: coil (a), globule (b), multiphase separation (c) and multistability (d). Snapshots result from full numerical simulations of the system. (**B**) Contact map (left), joint-probability distribution function for the root mean squared distance (r.m.s.d.) *d*_A_ between A-monomers and the r.m.s.d. *d*_B_ between B-monomers (center), and typical time-evolution of *d*_A_ and *d*_B_ along one simulated trajectory (right), obtained from full numerical simulations for a parameter set inside the multistability region (*d*_2_ in (A)). Time is given in arbitrary simulation time-unit (see Supplementary Notes).

**Figure 3.**
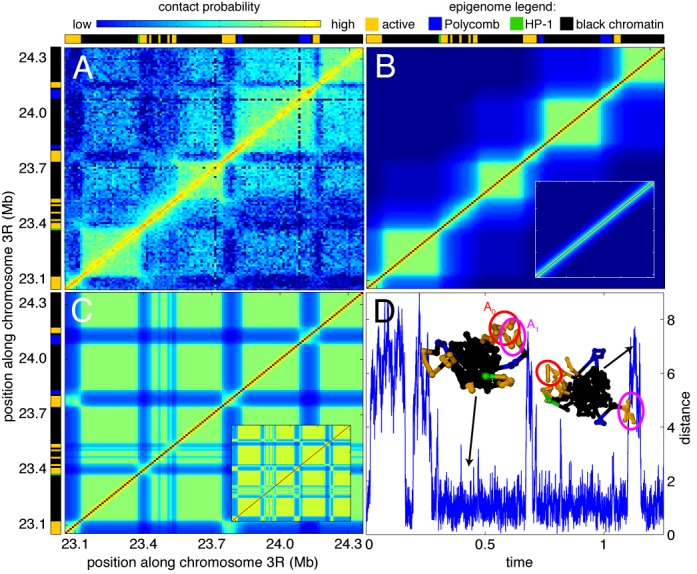
(**A**) Experimental Hi-C contact map for the chromatin region located between 23.05 and 24.36 Mb of chromosome 3R (from (11)). Epigenetic domains (from (1)) are given at the top and at the left borders of the figure: active (orange), Polycomb (blue), HP-1 (green) and black chromatin. (B and C) Examples of predicted contact maps inside the multistability region (*U*_ns_ = −25 *k*_B_*T*, *U*_s_ = −63 *k*_B_*T*) starting from a coil (**B**) or a MPS (**C**) configuration (see insets). (**D**) Time-evolution of the distance between the centers of masses of the active domains A_0_ and A_1_ along one simulated trajectory. Insets represent typical conformations of the chain.

**Figure 4.**
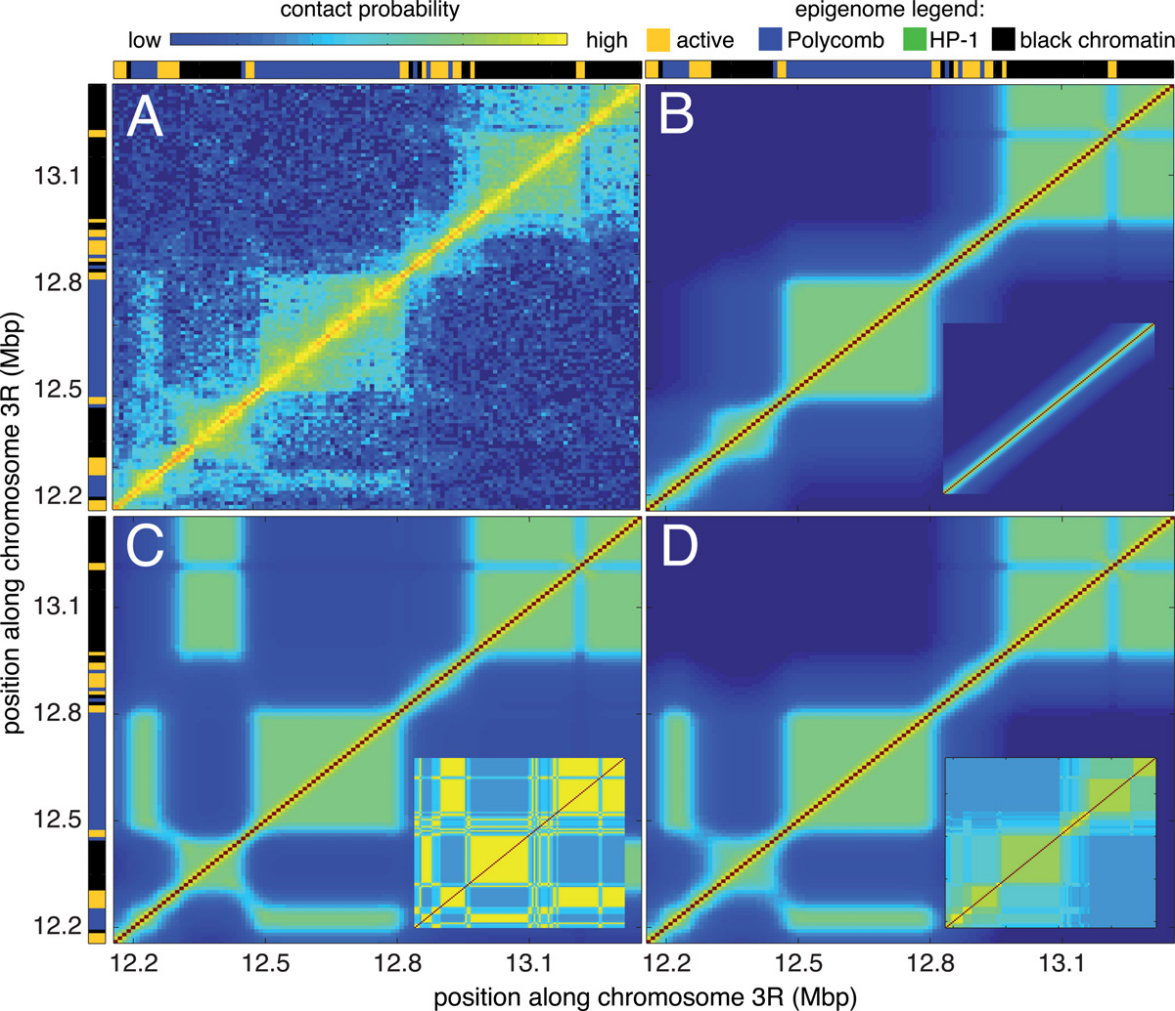
(**A**) Experimental Hi-C contact map for the chromatin region located between 12.16 and 13.36 Mb of chromosome 3R (from (11)). Epigenetic domains (from (1)) are given at the top and at the left borders of the figure: active (orange), Polycomb (blue), HP-1 (green) and black chromatin. (B, C and D) Examples of predicted contact maps inside the multistability region (*U*_ns_ = −40 *k*_B_*T*, *U*_s_ = −44 *k*_B_*T*) starting from a coil (**B**), a MPS (**C**) or a experimental-like (**D**) configuration (see insets).

Altogether, these experimental data reveal a clear link between the epigenome, the 3D chromosome architecture and the gene transcription pattern. However, it is still unclear what are the mechanisms behind 1D epigenome assembly and its 3D folding in TADs, and how these 1D and 3D organizations precisely contribute to gene regulation. Here, we address the question of the 3D folding of the epigenome using physical modeling. In particular, we ask if it is possible to interpret the observed correlations between the epigenome and the 3D chromatin organization in TADs by means of polymer physics arguments.

A generic and minimal model to investigate the large-scale 3D organization and dynamics of chromosomes is to consider chromatin as a semi-flexible self-avoiding homopolymer (19). The conformation of such polymer is controlled by the interplay between thermal motion, steric repulsion and effective monomer–monomer interactions that may account for steric confinement, attractive or repulsive interactions between monomers or interactions mediated by the solvent. For example, in a ‘good solvent’ condition, when steric repulsion dominates, the chain adopts a swollen coil state (20). In a ‘poor solvent’ condition, when effective attraction prevails, the polymer chain becomes compressed and equilibrates into a collapsed globular phase characterized by a high monomer density and a contact frequency that is almost constant at large separation distance. Between these two conditions, at the transition point (the so-called *θ*-temperature) where repulsion and attraction counterbalance, the chain adopts a Gaussian coil state. Such homopolymeric model with appropriate geometric constraints provide a fairly good description of large-scale conformational properties from yeast to human and in particular a good fit of different scaling behaviors like the dependence of the contact frequency or of the mean distance on the linear genomic distance. In yeast, static and dynamic studies suggest a brush-like equilibrated organization (21,22) of chromosomes with scaling properties compatible with a weakly collapsed state; in fly and human, where chromosomes are much longer, large-scale organization is characterized by a dense state that has been primarily associated to a non-equilibrium fractal globule composed of spatially segregated long-lived domains (23,24) but that can also be mapped to a semi-dilute solution of non-concatenated rings at equilibrium (25,26) or to a linear homopolymer with dynamic attractive self-interactions (27,28).

However, most of these models consider a homopolymer that cannot obviously account for the regional variability of the chromatin organization and in particular for the spatial compartmentalization of the epigenome. One has to introduce genomic and/or epigenomic specificities in the folding model (27,29–32). Here, we posit that chromatin folding is driven by effective epigenomic-dependent interactions between chromatin loci. To test this hypothesis, we propose a generic and new theoretical approach by treating chromatin as a block copolymer, where each block corresponds to an epigenomic domain and where each monomer interacts preferentially with other monomers of the same chromatin type. This is largely motivated by the observations of self-interactions between chromatin types (11) and is also supported by increasing evidence that some architectural proteins might promote physical bridging (33,34). We adapt a Gaussian self-consistent approach (35,36) to derive equilibrium phase diagram and contact maps for any block copolymers, as a function of two control parameters, the specific and non-specific monomer–monomer interaction. When considering block copolymers build from the epigenomic landscape of *Drosophila* (1), we show that this simple physical model accounts very well for the folding patterns in TADs observed in Hi-C experiments (11). As a main and very original outcome, this model provides a physical basis for the existence of multistability in chromosome organization. We show indeed how some experimental patterns are fully consistent with multistable conformations where TADs of the same epigenomic state interact transiently or long-lastly with each other.

## MATERIALS AND METHODS

### Block copolymer model

Chromatin is modeled as a self-avoiding bead-spring polymer containing *N* monomers, each monomer representing 10 kb of DNA. A given conformation of the chain is characterized by a Hamiltonian *H*. The Hamiltonian is made of two contributions *H* = *H*_chain_ + *H*_inter_ where *H*_chain_ describes the self-avoiding Gaussian chain and *H*_inter_ accounts for attractive short-range interactions between monomers. Connectivity between successive beads is modeled by a spring and the self-avoidance by a repulsive hard-code potential. The self-avoiding Gaussian chain is then described by

(1) \begin{equation*} H_{{\rm chain}} = \frac{{3k_{{\rm B}} T}}{{2l^2 }}\sum\limits_n {({\boldsymbol X}_n - {\boldsymbol X}_{n - 1} )^2 } + \sum\limits_{n < m} {U_{{\rm hc}} (r_{nm} )} \end{equation*}

with ***X****_n_* the position of monomer *n*, *l* the bond length of the pure Gaussian chain, *r_nm_* the distance between monomers *n* and *m*, and *U*_hc_(*r_nm_*) a truncated Lennard–Jones like potential (see Supplementary Notes). Contact interactions between monomers are modeled by a Gaussian-like potential:

(2) \begin{equation*} H_{{\rm inter}} = \sum\limits_{n < m} {E_{nm} \exp \left[ { - \frac{{r_{nm}^2 }}{{2r_0^2 }}} \right]} \end{equation*}

with *E_nm_* the strength of interaction between monomers *n* and *m*, *r*_0_ defining the length-range of the interaction. We note that the effects described in the article are robust over different parameter values, Hamiltonian forms and size of the investigated region (see Supplementary Figures S1 and S2).

### Gaussian self-consistent approximation

To simplify, the stochastic dynamics of the chain is modeled by a set of coupled Langevin equations (37):

(3) \begin{equation*} \xi \frac{{d{\boldsymbol X}_n }}{{dt}} = - \frac{{\partial H}}{{\partial {\boldsymbol X}_n }} + {\boldsymbol \eta} _n (t),\quad n = 1, \ldots ,N \end{equation*}

with *ξ* the friction coefficient and ***η****_n_* delta-correlated white noises that accounts for stochastic fluctuations of the system. The probability distribution function of *Y* = {***X**_n_*} verifies therefore the Fokker–Planck equation (38):

(4) \begin{equation*} \frac{{\partial P}}{{\partial t}} = \frac{1}{\xi }\sum\limits_n {\left[ {\frac{\partial }{{\partial {\boldsymbol X}_n }}\left( {P\frac{{\partial H}}{{\partial {\boldsymbol X}_n }}} \right) + k_{\rm B} T\frac{{\partial ^2 P}}{{\partial {\boldsymbol X}_n^2 }}} \right]} \end{equation*}

At each time point, we approximate *P* by a multivariate Gaussian distribution *P*(*Y*, *t*) ≈ (1/*Z*)exp[−*Y^+^C*(*t*)^−1^*Y/*2] with *C*(*t*) = {〈***X**_n_·**X**_m_*〉/3} the covariance matrix of *Y* at time *t*. To find an evolution equation for *C*, we adapt the approach developed by Ramalho *et al.* for biochemical reaction networks (36) to polymer dynamics: starting from the Gaussian distribution at time *t*, we can estimate with Equation (1) the evolved distribution at *t + δt*, then we look for the Gaussian distribution that better describes this evolved distribution in terms of relative entropy, Kullback–Leibler divergence or Gibbs free energy. This leads to (see Supplementary Notes)

(5) \begin{equation*} \xi \frac{{dC}}{{dt}} = {\langle} J {\rangle} C + C {\langle} J^ + {\rangle} + N_{\rm r} \end{equation*}

with *N*_r_ the covariance matrix of the random processes {***η****_n_*} and *〈J〉 = −∫dY P*(*Y*, *t*) (*∂*^2^*H)*/(*∂**X**_n_∂**X**_m_*), the average value (over the current Gaussian distribution) of the Hessian matrix of *H*. Since, by definition, the mean squared distance between monomer *m* and *n* is given by *D_mn_* *=* 〈(***X**_m_ − **X**_n_*)^2^〉/3 = *C_mm_* + *C_nn_* − 2*C_mn_* and *〈J〉* is a function of {*D_mn_*} (see Supplementary Notes), Equation (5) leads directly to the self-consistent equation:

(6) \begin{eqnarray*} &&\xi \frac{{dD_{mn} }}{{dt}} = 4k_{\rm B} T\nonumber \\ &&- \sum\limits_k {( {\langle} J_{mk} {\rangle} - {\langle} J_{nk} {\rangle} )(D_{mk} - D_{nk} )} \end{eqnarray*}

where *k*_B_*T* is the typical amplitude of stochastic fluctuations. Note that Timoshenko *et al.* have derived a similar equation for copolymers using Gibbs–Bogoliubov inequality (35).

Equation (6) is solved by numerical integration: starting from different initial conditions, we use a fifth order adaptive Runge–Kutta algorithm (39) until a stationary regime is achieved.

### Numerical simulations

Full numerical simulations of the block copolymer model were performed using a home-made molecular dynamics program. Integration of the trajectories was implemented using a standard velocity-Verlet algorithm and thermalization of the system was carried out by an Andersen thermostat (40) (see Supplementary Notes). Starting from a random configuration, we first let the system reach equilibrium before taking measurements on the system. We note that the results on the dynamics of the multistability region are robust over different stochastic collision frequencies of the thermostat (see Supplementary Figure S3).

## RESULTS

### Modeling chromatin as a block copolymer

The 1D sequence of epigenomic domains along the polymer chromatin allows the mapping between the system of interest and a block copolymer. Block copolymers are heteropolymers composed by successive blocks of different monomers. They have been intensively studied in the fields of polymer physics and chemistry and exhibit many remarkable properties, like their ability to form structured spatial conformations very similar to chromatin organization in the nucleus. In this paper, we propose a minimal extension of the homopolymeric model by considering chromatin as a block copolymer with interaction terms that would depend on the local epigenomic state. This introduces immiscibility between the monomers of different types that is well known to induce phase separation (20).

We model the chromatin fiber as an interacting self-avoiding bead-and-spring chain. Each monomer represents a portion of DNA (10 kb) and is characterized by an epigenetic state (see Figure 1). In addition to standard excluded volume interactions, we consider attractive short-range interactions *E_mn_* between monomers. In the following, we assume only two types of interactions: (i) non-specific interactions *U*_ns_ between every pairs of monomers that effectively account for compaction effect due to confinement into the nucleus; and (ii) specific attractive interactions *U*_s_ between monomers having the same epigenetic state (Figure 1). Hence *E_mn_ = U*_ns_ *+ δ_mn_U*_s_ with *δ_mn_* = 1 (resp. 0) if monomers *m* and *n* have (resp. have not) the same epigenetic state.

This specificity is motivated by many experimental evidence suggesting effective interactions between loci of identical chromatin state. Indeed, it has been shown that Polycomb group (34,41,42) or HP1 (33) proteins may create physical bridges between distant heterochromatin regions. Furthermore, mutualization of transcription machinery resources or DNA looping mediated by architectural proteins like cohesin and mediator may also lead to effective attractions between active loci (9,10,12,43,44). And, black chromatin is often associated with lamins (1) suggesting effective interactions mediated by the nuclear lamina. For simplicity, we assume that all the specific interactions have the same strengths (i.e. for example two Polycomb-like monomers interact with the same intensities than two HP1-like monomers).

While numerical simulations of such stochastic system could be time demanding, we develop a Gaussian self-consistent approximation that allows a fast scanning of the parameter space and that enables the efficient computation of expectation values for the HiC-map of the copolymer. This type of approach has already proven to be useful and accurate when studying copolymers (35,45). At each time point, the probability distribution function of the chain conformation is approximated by a multivariate Gaussian distribution whose parameters are determined using the maximum entropy principle (36) (see ‘Material and Methods’ section for details). This approximation leads to a closed set of ordinary differential equations that describe the dynamics of the mean squared distances between monomers (Equation (6)). We solve this set of non-linear equations in the steady-state limit by numerical integration. From the computed squared distances *D_mn_*, we estimate the probability of contact *P_mn_* between two monomers using the Gaussian approximation *P_mn_* ≈ *AD_mn_*^−3/2^ (with *A* a constant numerical factor). It has to be noted that the self-consistent approach can only give an approximate solution of chromatin folding due to the rough treatment of excluded volume. To verify that our conclusions are not artifacts of the approximation, we also perform for some parameter sets full numerical simulations of the copolymer using molecular dynamics (see ‘Material and Methods’ section). Moreover, we make the assumptions that the polymer chains are equilibrated. Detailed simulations of confined self-avoiding homopolymers (24) have suggested that strong topological constraints may slow down the chain dynamics such that equilibration of very long confined chains can be extremely long (24), well above the cell cycle length. However, recent theoretical studies (26) have estimated that below few Mb, the typical length scale investigated in this work, topological confinement is negligible and polymer chains may safely be considered as equilibrated.

### Heterogeneous chromatin exhibits a complex phase diagram with multistability

To illustrate the generic effects predicted by the model, we consider, as a toy example, a chain of 120 beads with an alternation of active (A) and black (B) epigenetic domains of the same size ((A_10_B_10_)_6_). Figure 2 shows the richness and complexity of the observed behaviors as we vary the strengths of compaction (via *U*_ns_) and of specificity (via *U*_s_) even for such simple epigenetic sequence. The phase diagram is made of four different regions. For weak compaction and specificity, the system is in a coil phase with extended chain conformations. As we increase the compaction at weak specificity, the chain undergoes a *θ*-collapse transition to a globular compact phase. For strong compaction and specificity, we observe checkerboard-like contact map characteristics of microphase separated (MPS) conformations where all monomers of the same epigenetic state are densely packed into distinct 3D domains. This phase is closed to the intermingled phase observed by Jerabek and Heermann when they introduce a sinusoidal binding affinity in their dynamic loop model (30). Between the coil and MPS phases, lies a region of multistability where Equation (6) has multiple fixed points depending on the initial conditions. Theses solutions are metastable intermediate configurations between coil and MPS. For example, heat-map d1 in Figure 2 represents pearl-necklace conformations where epigenetic domains have internally collapsed but remain isolated from each other, forming topologically associated domains. The size and location of the multistability region depend on the properties of the copolymer like the number of blocks, the number of block types, or the linear organization of the blocks along the polymer. However, we qualitatively observe that the size of the region grows with the complexity of the epigenomic sequence (Supplementary Figure S4). The enlargement of the area goes often with an increase of the number of metastable states (35).

To go further in the characterization of the multistability, we perform full numerical simulations for sets of parameters inside the region of interest of the phase diagram. We observe that in most conformations the epigenomic domains are internally collapsed (Figure 2B, left). The dynamics of the chain is then composed by stochastic jumps between several families of metastable states (Figure 2B, right). Each family being characterized by transient contacts between two or more epigenomic domains of the same type that temporarily merge together (Figure 2B, center). Association and dissociation dynamics between domains depend on the position inside the multistability region with faster dynamics close to the boundary with the coil phase (Supplementary Figure S3).

### *Drosophila* Hi-C maps are compatible with multistability

In this section, we apply the block copolymer model to different chromatin regions of *Drosophila melanogaster*. These regions have been chosen to be representative of the generic features of experimental Hi-C contact maps and of *in vivo* chromatin organization. Recently, Filion *et al.* have characterized the epigenome of embryonic *Drosophila* cell line Kc167 via statistical analysis of Dam-ID profiles of several chromatin-associated proteins (1). They found five typical epigenomic states: two euchromatic states that differ at the level of gene functions, and three heterochromatic states: the constitutive heterochromatin enriched in HP-1 proteins, the facultative Polycomb heterochromatin enriched in genes implicated in differentiation and development, as well as the so-called black chromatin, the prevalent type of repressive chromatin. In the following, we will use the epigenomic data from Filion *et al.*, where for simplicity we merged the two active states into one single epigenomic type, as the primary sequence of copolymers and vary the non-specific and specific interaction strengths to draw phase diagram as in the toy example.

#### Microphase separation in black chromatin

We start with a chromatin region (located between 23.05 and 24.36 Mb of chromosome 3R) whose epigenetic state is composed of large black domains separated by short active domains (Figure 3A). Its experimental Hi-C map (Figure 3A) shows the internal folding of black and active domains with almost uniform intra-domain contact probabilities. Inter-domain contacts are numerous between black domains. We also observe long-range contacts between active domains. For example, the active region A_1_ around 23.77 Mb is able to loop out the compact globule formed by the neighbor black domains to contact another active domain A_0_ (around 23.10 Mb) located 600 kb apart. In the context of a copolymer, these experimental observations are consistent with MPS-like conformations. Using the self-consistent approximation, we study the phase diagram of the region (modeled by a chain of 131 beads with 10 kb per bead) (Supplementary Figure S4). Many stationary solutions exist, that are consistent with the experimental Hi-C map. They are all located within the multistability region (examples are given in Figure 3B and C). In particular, we were able to reproduce the pattern of inter-black domains interactions and some long-range contacts between active domains (Figure 3C). By scanning a small part of the multistability region using full simulations, we find sets of parameter that catch qualitatively the average polymeric behavior of the chain as well as the specificity of the experimental data (Supplementary Figure S5). Of note, this suggests that black chromatin forms a compact metastable globule that transiently dissociates, and that small active domains are expelled at the periphery of the globule (insets Figure 3D). This localization allows dynamic interactions between the active regions (Figure 3D).

#### Long-range contacts between Polycomb domains

As another example, we choose the chromatin region located between 12.16 and 13.36 Mb of chromosome 3R. The epigenetic state is composed of a succession of black and Polycomb (blue) domains separated by short active regions (Figure 4A). The Hi-C map (Figure 4A) is made of internally folded domains corresponding to the epigenetic domains. Of particular interest are the long-range contacts observed between the Polycomb domains centered around 12.23 Mb (P_1_) and 12.65 Mb (P_2_), and the looping of the small active region (around 13.22 Mb) out the dense globule of black chromatin (B_0_) where it is embedded in. To investigate if the copolymer model is able to describe the experimental observations, we generate the phase diagram of this region (modeled by a chain of 120 beads) (Supplementary Figure S4). We observe the same four regions with a larger multistability area, consistent with the higher complexity of the local epigenome. In this region, depending on the initial conditions, many fixed points may be found for a fixed set of parameters, characteristics of frustrated phases that become dominant in copolymers with random sequences of monomers (35). The lack of information on chromatin organization in fly during mitosis does not allow to well defined the ‘true’ initial conditions. Like the previous black chromatin region, we observe that experiments are consistent with the multistability region. However, starting from coil, globular or MPS conformations, we were not able to reproduce the full characteristics of the experimental data (Supplementary Figure S6). While the internal folding of epigenetic domains and the looping out of the small active region are well described, we fail to mimic the inter-domain contacts, notably the simultaneous presence of contacts between P_1_ and P_2_ and absence of contacts between B_0_ and the black domain (B_1_) centered around 12.39 Mb. However, if the initial condition mimics the experimental Hi-C maps (see Supplementary Notes), within a significant portion of the multistability region, the system converges to a metastable state very close to the observed data (Figure 4D). This underlines the importance of initial conditions in the epigenome folding and suggests possible memory effects with the maintenance of long-range contacts already present in the mitotic chromatin organization. An alternative possibility to the mitotic reminiscence of long-range interactions is the existence of heterogeneities in the strengths of specific interactions. Indeed, assuming that Polycomb–Polycomb interactions are stronger than intra-black–chromatin interactions also allows to recapitulate the experimental data (Supplementary Figure S7).

## DISCUSSION

### A simple theoretical framework for epigenome folding

In this article, we introduce a block copolymer model to investigate the folding properties of chromosomes as a function of the underlying epigenome. This model considers a single input, namely the experimentally derived epigenome that defines the primary sequence of the copolymer, and only two control parameters, the non-specific and the specific monomer–monomer interactions. The former accounts for a global compaction level, the latter for the effective attraction between monomers of same chromatin type. We used an efficient computational approach that allows us to derive expected contact maps and explore phase diagrams over a broad range of parameters. Remarkably, we show that such a minimal model can account for the main generic properties observed experimentally. Our approach provides a simple, tractable and attractive theoretical framework for interpreting the organization of the epigenome at sub-chromosomal scale (from 10 kb to few Mb) and in particular its spatial compartmentalization in TADs. Here, folding of the genome is assumed to be mainly driven by effective attractive interactions between chromatin elements of same epigenomic types. Self-association promotes internal folding and leads to spatial segregation and insulation of adjacent epigenomic domains without the need to introduce any bridging or anchoring activities at the TAD boundaries. Increasing specific attraction essentially leads to further compaction of the TADs and global confinement promotes cross-talk between TADs of the same epigenomic state. Compartmentalization might be a way of coordinating and reinforcing the functional output of genomic regions by colocalization and mutualization of the same specific regulators.

### Chromatin organization is multistable and dynamic

One of the main outcomes of the copolymer model is the existence of a multistability region inside the phase diagram where epigenomic domains fold into topologically associated domains that interact transiently with each other. The dynamics of these interactions depend on the strength of the specific interactions, but also on the sizes of the epigenomic domains. For example, small domains, like most of the epigenomically active domains, would exhibit fast and dynamic interactions, while bigger domains may form long-lived metastable interactions. Comparison with experimental Hi-C maps of *Drosophila* suggests that biological situations are consistent with this multistability. This implies that, *in vivo*, chromatin organization is being dynamically and stochastically remodeled while conserving local key features like the TADs. This prediction of the model is in perfect agreement with recent single-cell Hi-C experiments in mouse showing the conservation of TADs between cells and the high variability of inter-domain contacts (14). Stochasticity in long-range inter-TAD interactions may represent an important source of intrinsic noise that may play an important role on the co-regulation of distant genes (46). However, during development or differentiation, since noisy systems closed to criticality are very sensitive to external stimuli (47,48), this variability might be useful to respond efficiently to developmental cues that drive the colocalization of distant loci (49).

**Figure 5.**
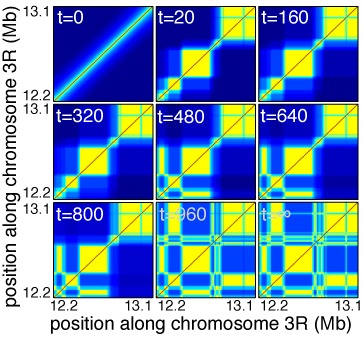
Evolution of the contact map for the chromatin region located between 12.16 and 13.36 Mb as a function of time *t* (in arbitrary simulation time-unit), starting from a coil-like conformation and ending at steady-state in a MPS-like conformation. Legend color as in Figure 4.

Recent experiments performed on senescent cells have shown the nuclear rearrangement of heterochromatic marks into non-overlapping micro-domains (18). Within our formalism, this suggests that chromatin organization may relax to microphase separation configuration in non-dividing cell. Figure 5 shows the dynamic evolution of a contact map predicted by the copolymer model starting from a coil state and ending in a MPS steady-state. Interestingly, we observe the very fast formation of TADs, followed by a long period of slow compaction where long-range interactions are gradually incorporated, until the copolymer experiences a very fast transition to MPS. This intermediate slowing-down is a signature of the glassy-like dynamics of copolymers when crossing the multistability/frustrated region (35). These predictions are also consistent with recent Hi-C experiments on synchronized HeLa cells (50) showing that the formation of TADs is already achieved in early G1 starting from a mitotic conformation where the organization in TADs is apparently lost, and that the Hi-C map remains fairly unchanged throughout the cell cycle except during mitosis. This suggests that in normal dividing cells, chromatin organization converges quickly to multistable conformations and does not have the time to relax to a MPS-like state due to the periodic reinitialization of the chromatin organization at mitosis.

### Toward inference and prediction

In this paper, we aimed at exploring the generic folding properties of the chromatin fiber. Therefore, for simplicity, we limit our approach to the simplest version of a block copolymer that we can build from the compartmentalization of the epigenome. The model assumes that all specific interactions have the same strength whatever the chromatin type and whatever the location along the genome. However, such two-parameter model needs to be refined in order to gain in predictability. In particular, the current model considers each domain as a homopolymer with uniform monomer–monomer interactions. This can only provide a coarse-grained understanding of epigenome folding and cannot account for variation in contact frequency and in particular for preferential pairwise (long-range) contact between discrete genomic loci. Recent studies (9,12) have proposed that these site- and lineage-specific contacts mediated by architectural proteins (insulators, cohesin and mediators) might indeed play a key role in the folding of chromosomes at the sub-Mb scale. Along the same line, anchoring at the membrane of particular sequence or/and epigenomic domains (via their association with lamina or nuclear pores) has been shown to be crucial for organizing chromatin inside the nucleus (30,51,52). In addition to the global, non-site-specific, interactions investigated in this study, focal large-scale looping and anchoring might indeed contribute to spatial compartmentalization of domains (11,12,53). Therefore, further improvements of the model will require to augment the number of parameters by allowing for variability of interaction at the monomer scale, and to infer specific interaction strengths that predict at best the observed contact maps.

The copolymer framework associated with the self-consistent Gaussian approximation may represent an efficient formalism to extract from the available experimental data the effective genomic and epigenomic interactions between chromatin loci (54). As a promising outcome of such inference process, would be a powerful tool to predict the chromatin organization in various conditions, allowing investigating *in silico* changes in TAD formations and long-range contacts when altering the epigenome. In particular, during development, cell differentiation proceeds by global and concomitant rearrangements of epigenomic profile, chromatin organization and transcriptional activity (49,55–57). Hence, our model may provide a very interesting framework for understanding how epigenome regulation (resp. deregulation) during development (resp. disease) could lead to cell phenotypic variations via large-scale chromatin reorganization.

## SUPPLEMENTARY DATA

Supplementary Data are available at NAR Online.

SUPPLEMENTARY DATA
