# Supplementary material for: Modeling epigenome folding: formation and dynamics of topologically associated chromatin domains
Source: Nucleic Acids Res. 2014 Aug 4;42(15):9553–61. doi: 10.1093/nar/gku698 (PMC4150797; doi:10.1093/nar/gku698)
Supplement: SUPPLEMENTARY DATA [file supp_42_15_9553__index.html]

Modeling epigenome folding: formation and dynamics of topologically associated chromatin domains — Modeling epigenome folding: formation and dynamics of topologically associated chromatin domains — Modeling epigenome folding: formation and dynamics of topologically associated chromatin domains — SUPPLEMENTARY DATA 

# Modeling epigenome folding: formation and dynamics of topologically associated chromatin domains

## SUPPLEMENTARY DATA

**Files in this Data Supplement:**

- SUPPLEMENTARY DATA
