# Supplementary material for: Modeling epigenome folding: formation and dynamics of topologically associated chromatin domains
Source: Nucleic Acids Res. 2014 Aug 4;42(15):9553–61. doi: 10.1093/nar/gku698 (PMC4150797; doi:10.1093/nar/gku698)
Supplement: SUPPLEMENTARY DATA [file supp_gku698_nar-00879-z-2014-File007.pdf]

Supplemental Material

Modeling epigenome folding: formation and dynamics of topologically-associated chromatin domains

Daniel Jost*, Pascal Carrivain†, Giacomo Cavalli† and Cédric Vaillant*
* *Laboratoire de Physique, École Normale Supérieure de Lyon, CNRS UMR 5672, Lyon, France*
† *Institute of Human Genetics, CNRS UPR 1142, Montpellier, France*

Contents

1 Supplementary Figures **2**

2 Supplementary Notes **9**
- 2.1 Block copolymer model 9
- 2.2 Gaussian self-consistent approximation 9
  - 2.2.1 Gaussian approximation and self-consistency 9
  - 2.2.2 Application to the block copolymer model 11
  - 2.2.3 Numerical integration 12
  - 2.2.4 Equivalence with the self-consistent method of Timoshenko, Kuznetsov and Dawson 12
- 2.3 Numerical simulations 13

1 Supplementary Figures

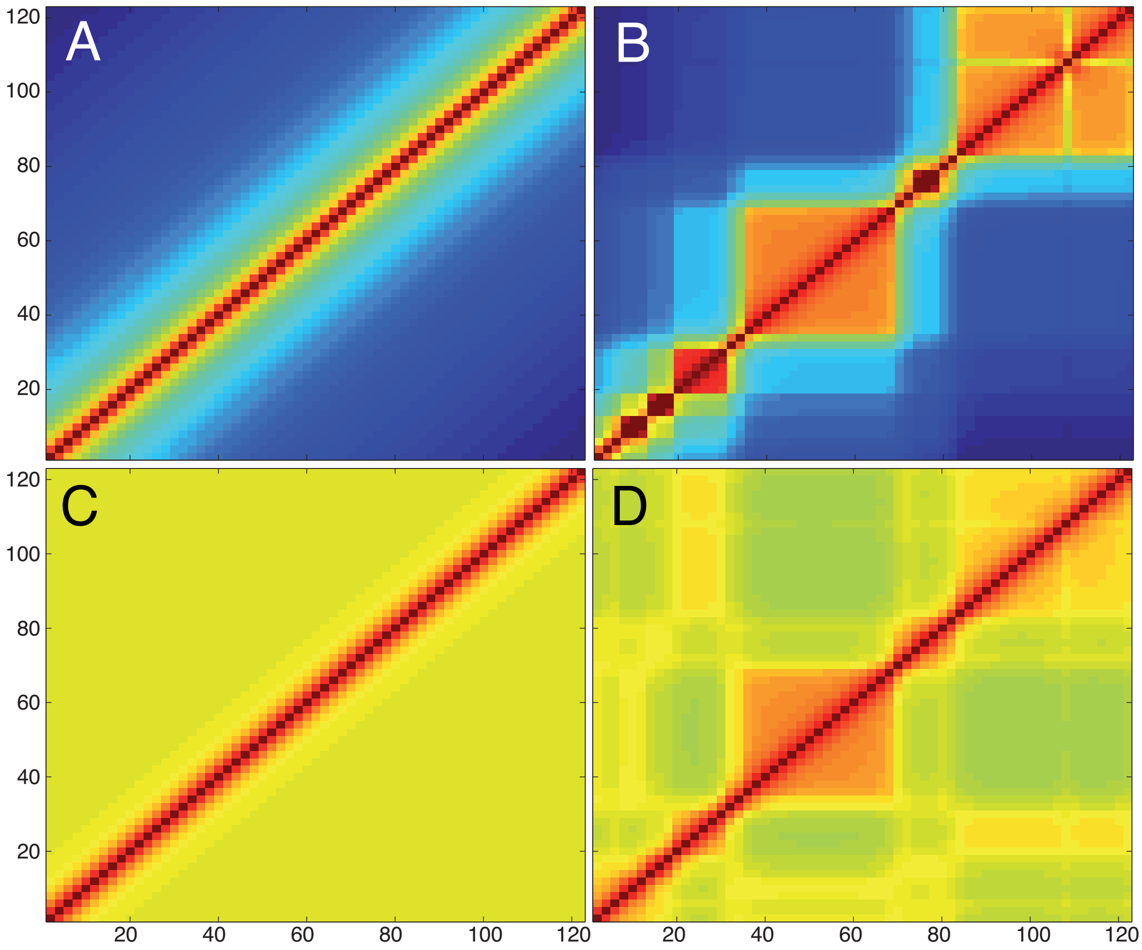

Fig.S 1: Typical computed contact maps (A: coil, B: pearl-necklace-like, C: globular, D: MPS-like) of the chromatin region located between 12.16 and 13.36 Mbp starting from a coil, predicted by the Gaussian self-consistent approximation but for a different form of the Hamiltonian. Like in Timoshenko *et al.* (1998), we considered here virial-type expansion contact interactions to account for hard-core repulsion and attraction between monomers: $H = (3k_BT/2l^2)\sum_n(\mathbf{X}_n - \mathbf{X}_{n-1})^2 + \sum_{n<m}(U_{ns} + U_s\delta_{n,m})\boldsymbol{\delta}(\mathbf{X}_n - \mathbf{X}_m) + \sum_{n\neq m\neq k} U_{hc}\boldsymbol{\delta}(\mathbf{X}_n - \mathbf{X}_m)\boldsymbol{\delta}(\mathbf{X}_m - \mathbf{X}_k)$ where $\boldsymbol{\delta}$ is the Dirac Delta function.

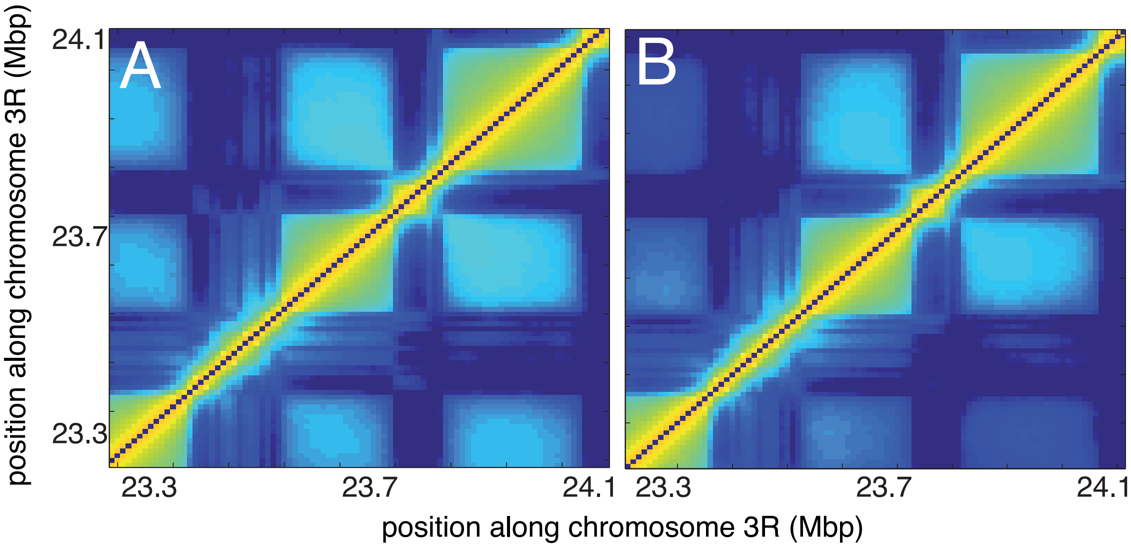

Fig.S 2: Predicted contact map of the chromatin region located between 23.23 and 24.13 Mbp of chromosome 3R when simulated alone (B) or embedded in a larger context (A, taken from Fig.S5C by zooming to the corresponding area) for the same set of parameters. A priori, the contact map of a region should depend on the size of the chain and on the primary sequence of the neighboring chromatin. We remark that the pattern of interactions itself is not affected by the size of the investigated region. We observe only weak alterations in the long-range intensities with domains located at the extremities of the chain. In our example, this is due to the absence during the simulations (in B) of a significant part of the black chromatin that contribute in stabilizing the black chromatin globule mentioned in Fig.3D of the main text.

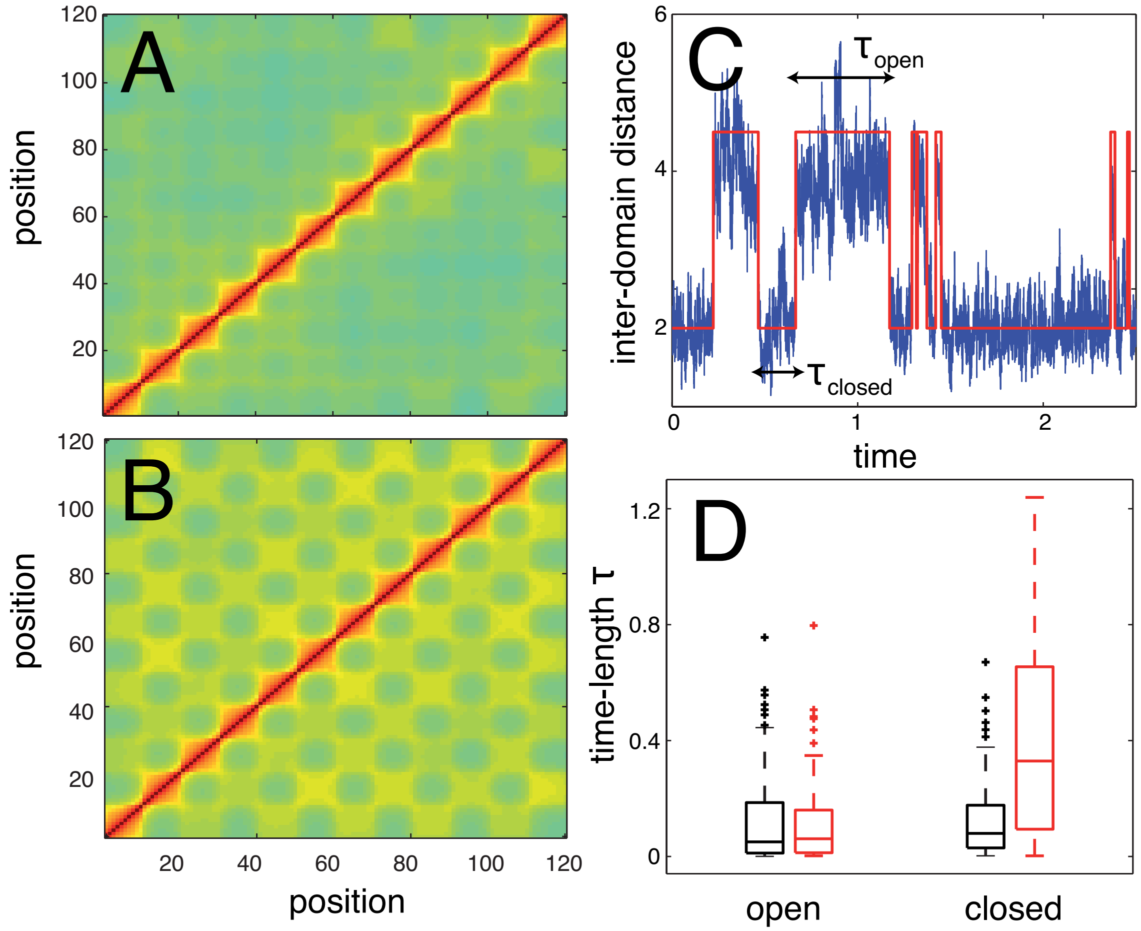

Fig.S 3: (A,B) Contact maps of the copolymer $(A_{10}B_{10})_6$ estimated from full numerical simulations for two parameter sets inside the multistability region: close to the boundary with the coil region (A) or with the microphase region (B). (C) Typical time-evolution of the root mean squared distance d between monomers of the same epigenomic state (blue line). Conformations can be classified between two categories: open ($d > 3$) or closed ($d < 3$). (D) Boxplots for the distribution of residence time in the open or closed states for (A) (black) or (B) (red). The lifetime of the transient contacts between TADs depends on the position inside the multistability region with shorter-lived contacts close to the coil phase (for (A): $\langle\tau_{\text{open}}\rangle = 0.13 \pm 0.02$, $\langle\tau_{\text{closed}}\rangle = 0.13 \pm 0.02$; for (B): $\langle\tau_{\text{open}}\rangle = 0.12 \pm 0.02$, $\langle\tau_{\text{closed}}\rangle = 0.40 \pm 0.04$). Qualitatively, this observation does not depend on the stochastic collision frequency used to simulate the heat bath. For a doubled frequency (= 10), we find for (A): $\langle\tau_{\text{open}}\rangle = 0.25 \pm 0.03$, $\langle\tau_{\text{closed}}\rangle = 0.19 \pm 0.02$; for (B): $\langle\tau_{\text{open}}\rangle = 0.14 \pm 0.02$, $\langle\tau_{\text{closed}}\rangle = 0.44 \pm 0.04$.

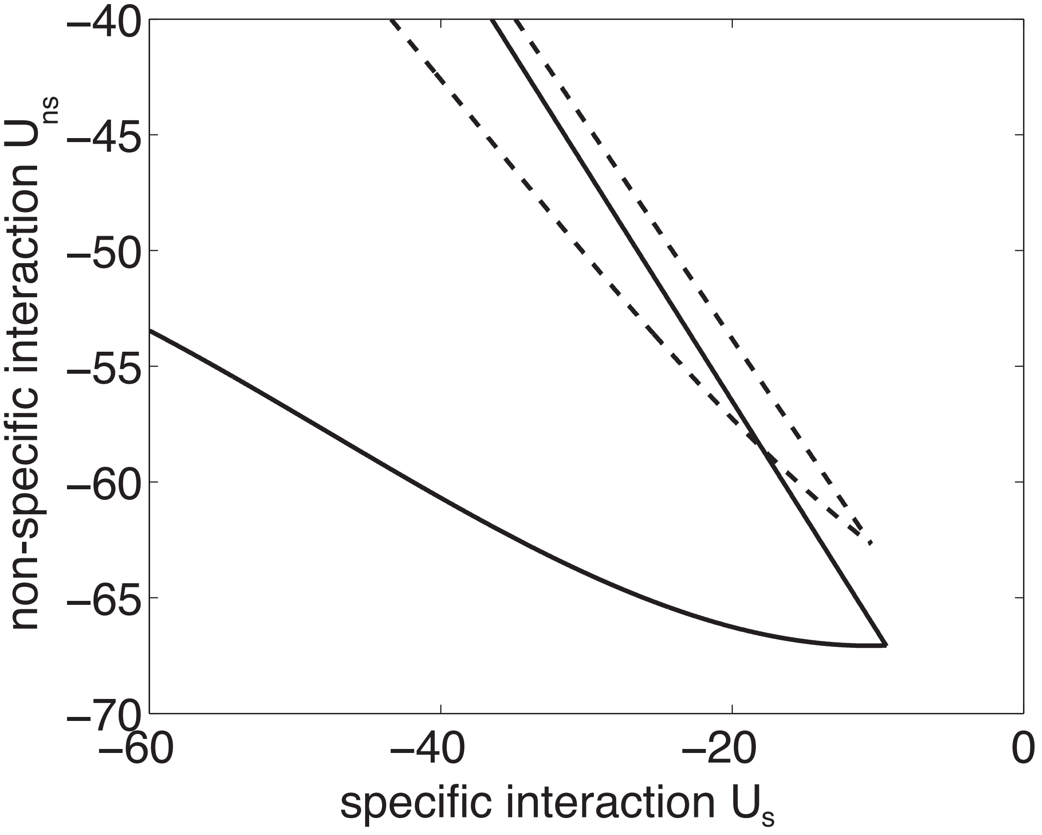

Fig.S 4: Boundaries of the multistability region for the regions located between 12.16 and 13.36 Mbp (full lines) and between 23.06 and 24.36 Mbp of chromosome 3R. The first chromatin region has a more complex pattern of epigenomic state leading to a larger multistability region.

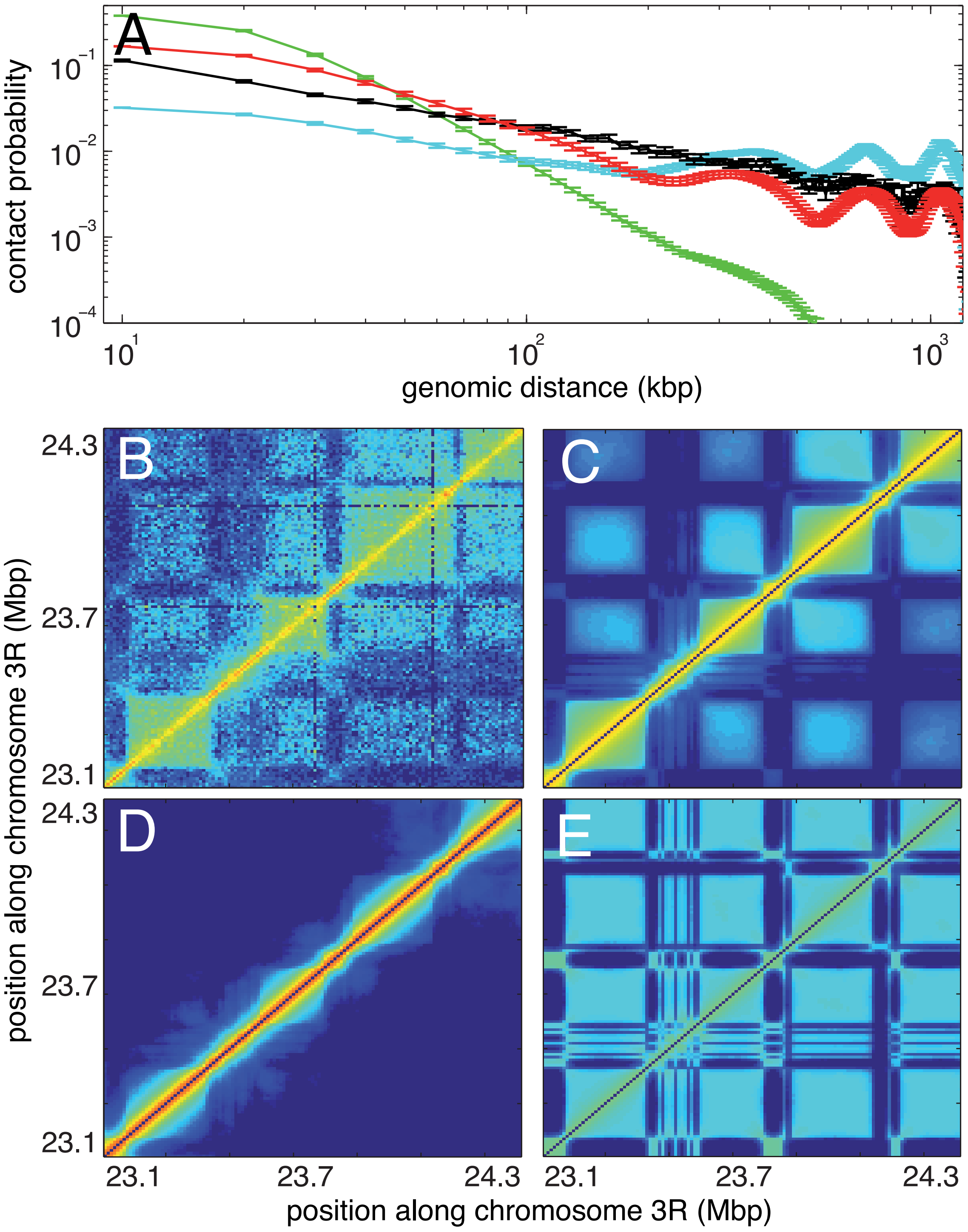

Fig.S 5: (A) Average contact probability between two loci of the chromatin region located between 23.05 and 24.36 Mbp of chromosome 3R, as a function of their genomic distance. (B,C,D,E) Steady-state contact maps. Taken from experimental data (black, B) or from full numerical simulations for parameter sets at the multistability/coil boundary (green, D), at the multistability/MPS boundary (cyan, E) or inside the multistability region (red, C). Legend color as in Fig. 3 of the main text.

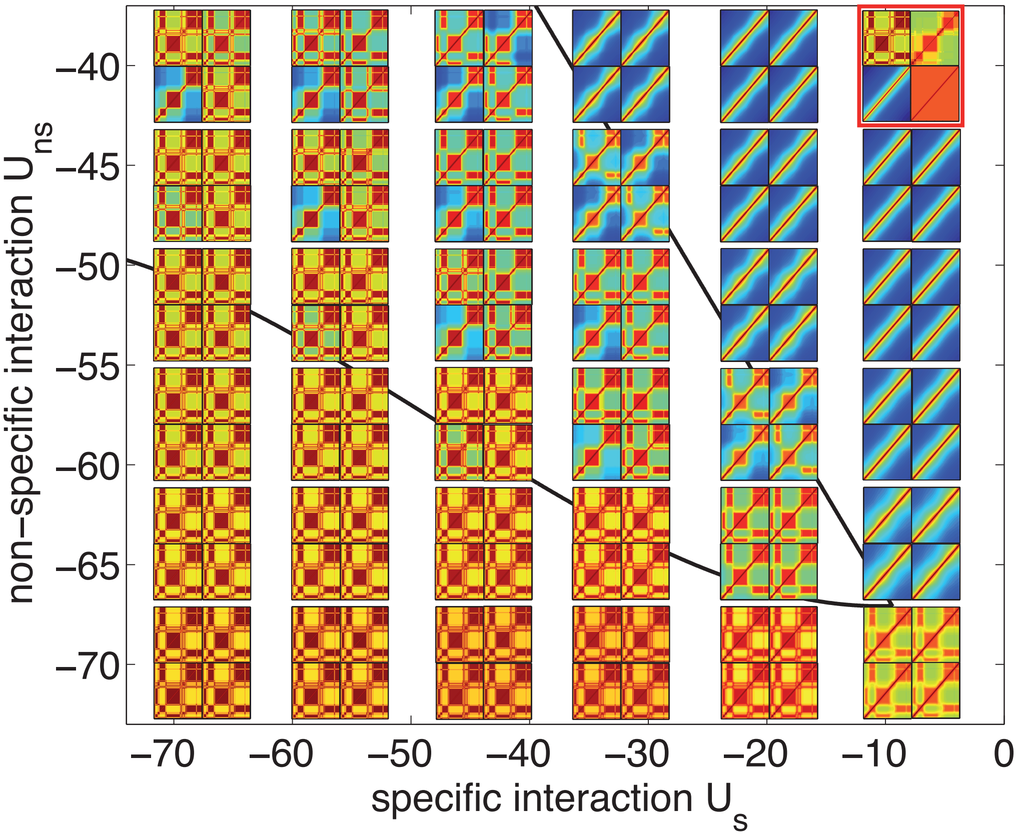

Fig.S 6: Steady-state contact maps of the chromatin region located between 12.16 and 13.36 Mbp of chromosome 3R predicted by the Gaussian self-consistent approximation as a function of the strength of specific and non-specific interactions, starting from a coil, a MPS, a globular or an experimental-like conformations (initial contact maps are drawn at the top right corner). In the multistability region, depending on the initial conditions, many different steady-state solutions can be found by the Gaussian self-consistent method, the true thermodynamic averages being a weighted sum of these different solutions.

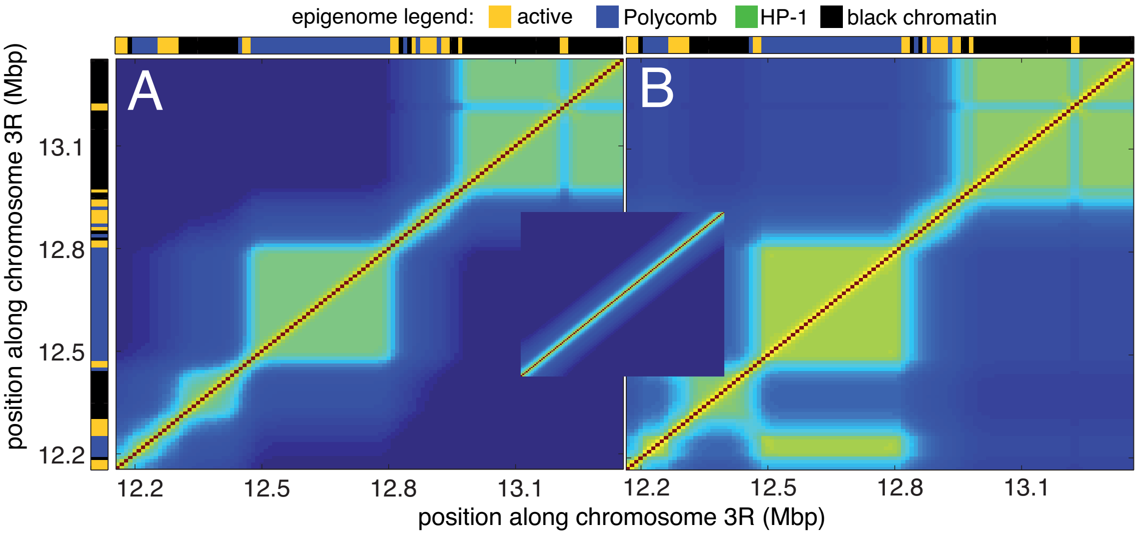

Fig.S 7: Predicted contact maps of the chromatin region located between 12.16 and 13.36 Mbp of chromosome 3R, starting from a coil, for homogeneous specific parameters (A) ($U_{ns} = -40k_BT$, $U_s = -44k_BT$) and when Polycomb-Polycomb interactions are 1.2 times stronger than other specific interactions (B). Reinforcing Polycomb-Polycomb interactions allows to accurately describe the experimental contact map without the spurious contacts between the two black domains B0 and B1.

2 Supplementary Notes

2.1 Block copolymer model

In this section, we describe in greater details the block copolymer model.

We model the chromatin fiber as an interacting self-avoiding bead-and-spring chain containing N monomers. Each monomer represents 10 kbp of DNA and is characterized by an epigenetic state. The Hamiltonian of the system $H = H_{chain} + H_{inter}$ is made of two contributions:

- H_{chain} the Hamiltonian of the self-avoiding chain, given by

$$H_{chain} = \frac{k}{2} \sum_{n=2}^{N} (\mathbf{X}_n - \mathbf{X}_{n-1})^2 + \sum_{n<m} U_{hc}(r_{n,m}) \tag{1}$$

  with $\mathbf{X}_n$ the position of monomer n, $k = 3k_B T/l^2$ (l the segment length of the pure Gaussian chain), U_{hc} the pair-wise repulsive hard-core potential and $r_{n,m}$ the relative distance between monomer n and m ($r^2_{n,m} = (\mathbf{X}_n - \mathbf{X}_m)^2$). U_{hc} is modeled by a truncated Lennard-Jones-like potential:

$$U_{hc}(r) = \begin{cases} U^0_{hc} \left[\left(\frac{\sigma}{r}\right)^{5/2} - \left(\frac{\sigma}{r}\right)^{5/4} + \frac{1}{4} \right] & \text{for } r \leq 2^{4/5}\sigma \\ 0 & \text{for } r > 2^{4/5}\sigma \end{cases}$$

  The motivation behind the exponents 5/2 and 5/4 is to insure integrability of the hard-core potential needed by the self-consistent approximation (see below). The cut-off occurs at the minimum of the potential to insure continuity. We choose $U^0_{hc} = 20k_B T$ and $2^{4/5}\sigma = l$.

- H_{inter} accounts for attractive short-range interactions between monomers and is made of non-specific interactions modeling compaction effect due to confinement, and of specific interactions modeling attraction between monomers having identical epigenetic states. H_{inter} is given by

$$H_{inter} = \sum_{n<m} (U_{ns} + U_s \delta_{e_n,e_m}) \exp[-r^2_{n,m}/(2r_0^2)] \tag{2}$$

  with U_{ns} and U_s the strength of non-specific and specific interactions, $\delta_{e_n,e_m} = 1$ (0) if the epigenetic states e_n and e_m of monomers n and m are equal (or not), and r_0 the typical length-scale of the short-range interaction. The motivation behind this Gaussian-like potential is that the number of contacts between two Gaussian chains (the 10kbp-long subchains contained in each monomer) scales as $\exp[-d^2/(2r_0^2)]$ with d the distance between the centers of mass and r_0 the typical radius of gyration of the subchain. We choose $r_0 = l/\sqrt{6}$.

2.2 Gaussian self-consistent approximation

In this section, we detail the self-consistent approximation used to derive Eq.2 of the main text.

2.2.1 Gaussian approximation and self-consistency

The dynamics of the chain is described by a set of Langevin equations

$$\xi \frac{d\mathbf{X}_n}{dt} = -\frac{\partial H}{\partial \mathbf{X}_n} + \boldsymbol{\eta}_n(t) \quad n = 1, ..., N \tag{3}$$

with ξ the friction coefficient and η_n delta-correlated white noise ($\langle \boldsymbol{\eta}_n \rangle = 0$ and $\langle \eta^\alpha_n(t) \eta^\beta_m(t') \rangle = 2\xi k_B T \delta(t-t') \delta_{n,m} \delta_{\alpha,\beta}$ with $\alpha, \beta \in \{x, y, z\}$).

From the set of Langevin equations, one can write the corresponding Fokker-Planck equation for $P(Y,t)$ the probability distribution function (p.d.f) of the chain conformation $Y = \{\mathbf{X}_n\} = \{X_1^x, X_1^y, X_1^z, X_2^x, ...\}$

$$\partial_t P(\{\mathbf{X}_n\},t) = \frac{1}{\xi}\sum_n \left[\frac{\partial}{\partial \mathbf{X}_n}\left(P\frac{\partial H}{\partial \mathbf{X}_n}\right) + k_B T \frac{\partial^2 P}{\partial \mathbf{X}_n^2}\right] \quad (4)$$

At each time point, we approximate P by a multivariate Gaussian distribution

$$P \approx \frac{1}{(2\pi)^{3N/2}|\det(\bar{C})|^{1/2}} \exp\left[-\frac{1}{2}(Y-\bar{Y}(t))^\dagger \bar{C}(t)^{-1}(Y-\bar{Y}(t))\right] \quad (5)$$

with $\bar{Y}(t)$ ($\bar{Y}_{i,\alpha} = \langle X_i^\alpha\rangle$) and $\bar{C}(t)$ ($\bar{C}_{i,\alpha;j,\beta} = \langle X_i^\alpha X_j^\beta\rangle$) the first and second moment of the Gaussian. Isotropy of the system imposes already $\bar{Y}=0$ and that $\bar{C}_{i,\alpha;j,\beta} = C_{i,j}\delta_{\alpha,\beta}$ with $C_{i,j} = \langle \mathbf{X}_i \cdot \mathbf{X}_j\rangle/3$.

In the next, we aim to derive an equation that describes the dynamics of C using the approach developped by Ramalho et al. for approximating p.d.f dynamics but in the context of biochemical reaction networks {Ramalho et al, Phys. Rev. E, **87**: 022719 (2013)}. The general idea is to assume an initial Gaussian distribution for Y, then to evolve it according to the Fokker-Planck equation, and then find the Gaussian distribution that best fits it. Let's first focus on the deterministic part of this evolution (by putting $k_BT = 0$) before adding the stochastic noise. Consider that we have a Gaussian distribution $P(Y)$ at time t (with a covariance $C(t)$). After an infinitesimal time step dt, the evolved (deterministic) p.d.f will be

$$P_e(Y') = P(Y)\left|\frac{dY}{dY'}\right| = \frac{P(Y)}{|\det(I + dtJ/\xi)|} \quad (6)$$

with $Y' = Y + dt(-\partial H/\partial X_n)/\xi$ and $J = -(\partial^2 H)/(\partial X_n \partial X_m)$ the Jacobian of the (deterministic part of the) Langevin equations (the opposite Hessian of the Hamiltonian H). Due to the non-linearity of $(-\partial H/\partial X_n)$, P_e is no longer a Gaussian. However, we aim to determine the closest Gaussian distribution P' (characterized by a covariance C') to P_e in term of information content using the maximum entropy principle. One requirement of this principle is to minimize the Kullback-Leibler divergence of P' to P_e defined as

$$d_{KL}(P'||P_e) = \int dY' P'(Y') \log\left(\frac{P'(Y')}{P_e(Y')}\right) \quad (7)$$

Minimization of $d_{KL}(P'||P_e)$ with respect to C' leads to {Ramalho et al, Phys. Rev. E, **87**: 022719 (2013)}

$$C' = C + dt(\langle J\rangle C + C\langle J\rangle^\dagger)/\xi \quad (8)$$

with $\langle J\rangle$ the average value of J over the Gaussian distrution $P(Y)$. We now consider the effect of intrinsic fluctuations. Over dt, the evolution Y'' of Y is given by Y' augmented by the random variable ηdt which has a Gaussian distribution with covariance Ndt/ξ ($Y'' = Y' + \eta dt/\xi$). Therefore the evolved p.d.f with noise is given by the convolution

$$\begin{aligned} P(Y'') &= \int d\eta \left(\frac{\exp[-(Y''-\eta)^\dagger C'^{-1}(Y''-\eta)/2]}{Z'}\right)\left(\frac{\exp[-\eta^\dagger(Ndt)^{-1}\eta/2]}{Z_\eta}\right) \\ &= \frac{\exp[-Y''^\dagger(C'+Ndt/\xi)^{-1}Y''/2]}{Z''} \end{aligned} \quad (9)$$

Therefore after dt, the closest Gaussian distribution of the evolved p.d.f is characterized by the covariance matrix $C'' = C' + Ndt/\xi = C + dt(\langle J\rangle C + C\langle J\rangle^\dagger + N)/\xi$. Taking the limit $dt \to 0$ leads to

$$\xi \frac{dC}{dt} = \langle J\rangle C + C\langle J\rangle^\dagger + N \tag{10}$$

This equation is formally very similar to the linear noise approximation (LNA) {van Kampen. Stochastic Processes in Physics and Chemistry, North-Holland (2001)} but with the significant difference that, in Eq.10, J is average over the current Gaussian distribution while in the LNA J is evaluated at the average value of Y.

2.2.2 Application to the block copolymer model

By definition of J, we find

$$J_{n,m} = -\frac{\partial^2 H}{\partial X_n \partial X_m} = \begin{cases} (X_n - X_m)^2 \frac{1}{r_{n,m}} \frac{\partial}{\partial r_{n,m}} \left(\frac{1}{r_{n,m}} \frac{\partial U_{n,m}}{\partial r_{n,m}} \right) + \frac{1}{r_{n,m}} \frac{\partial U_{n,m}}{\partial r_{n,m}} & \text{for } n \neq m \\ -\sum_{k\neq n} J_{n,k} & \text{for } n = m \end{cases} \tag{11}$$

with $r_{n,m}$ and $U_{n,m}$ respectively the distance and the interaction potential between monomers n and m. $\langle J_{n,m}\rangle$ is then given by averaging over the current Gaussian distribution of $\mathbf{X}_n - \mathbf{X}_m$, ie, for $n \neq m$

$$\begin{aligned} \langle J_{n,m}\rangle &= \int \Bigg\{ 2\pi r_{n,m}^2 \sin\theta dr_{n,m} d\theta \left[(r_{n,m}\cos\theta)^2 \frac{1}{r_{n,m}} \frac{\partial}{\partial r_{n,m}} \left(\frac{1}{r_{n,m}} \frac{\partial U_{n,m}}{\partial r_{n,m}} \right) + \frac{1}{r_{n,m}} \frac{\partial U_{n,m}}{\partial r_{n,m}} \right] \\ &\quad \times \frac{\exp[-r_{n,m}^2/(2D_{n,m})]}{(2\pi D_{n,m})^{3/2}} \Bigg\} \end{aligned} \tag{12}$$

with $D_{n,m} = \langle (X_n - X_m)^2 \rangle = \langle (\mathbf{X}_n - \mathbf{X}_m)^2 \rangle / 3$, the third of the mean squared distance between n and m. Finally, we find for $n \neq m$

$$\begin{aligned} \langle J_{n,m}\rangle &= -k\left(2\delta_{n,m} - \delta_{n-1,m} - \delta_{n+1,m}\right) + \frac{r_0^3(U_{ns} + U_s \delta_{e_n,e_m})}{(D_{n,m} + r_0^2)^{5/2}} \\ &\quad + \frac{5\sigma^{5/4} U_{hc}^0}{2^{1/4} 12\sqrt{\pi}} \left(\frac{1}{D_{n,m}^{13/8}} \right) \Bigg[\frac{2\sigma^{5/4}}{D_{n,m}^{5/8}} \Big(\Gamma_{inc}[1/4, 2^{3/5}\sigma^2/D_{n,m}] - \Gamma[1/4] \Big) \\ &\quad + 2^{5/8} \Big(\Gamma[7/8] - \Gamma_{inc}[7/8, 2^{3/5}\sigma^2/D_{n,m}] \Big) \Bigg] \end{aligned} \tag{13}$$

with $\Gamma_{inc}[a,z] = \int_z^\infty t^{a-1} e^{-t} dt$ the incomplete gamma function. $\langle J_{n,n}\rangle = -\sum_{k\neq n} \langle J_{k,n}\rangle$.

From Eq.10, we derive a corresponding equation for D (Eq.2 of the maint text). Remarking that $D_{n,m} = C_{n,n} + C_{m,m} - 2C_{n,m}$, we find for $n \neq m$

$$\begin{aligned} \xi \frac{dD_{m,n}}{dt} &= N_{n,n} + N_{m,m} - 2N_{m,n} + (C_{m,m} - C_{n,n}) \sum_k (\langle J_{m,k}\rangle - \langle J_{n,k}\rangle) \\ &\quad - \sum_k (\langle J_{m,k}\rangle - \langle J_{n,k}\rangle)(D_{m,k} - D_{n,k}) \\ &= 4k_B T - \sum_k (\langle J_{m,k}\rangle - \langle J_{n,k}\rangle)(D_{m,k} - D_{n,k}) \end{aligned} \tag{14}$$

Since $\langle J\rangle$ is a fonction of D, the last equation is self-consistent and allows to compute the dynamics of the mean squared distance matrix.

2.2.3 Numerical integration

We choose k_BT as the unit of energy, l as the unit of length, and $\xi l^2/(k_BT)$ as the unit of time. The set of non-linear equations defined in Eq.14 is solved in the steady-state limit by numerical integration. For a given epigenetic pattern, starting from different initial conditions, we implement a fifth order adaptative Runge-Kutta algorithm {Press et al. Numerical Recipes, Cambridge University Press (2007)} to find the fixed points. For parameter sets located in the coil, globule or microphase regions (see Fig. 2 of the main text), the algorithm converges, independently of the initial condition, to a unique fixed point. In the multistability region, it exists several fixed points that corresponds to the stable and metastable thermodynamic states. The algorithm cannot give the relative weight of each state in the thermodynamic ensemble.

From the steady-state matrix D, in the Gaussian approximation, the probability $P_{m,n}$ of contact between monomers m and n is given by

$$P_{m,n} = \int_0^a 4\pi r^2 dr \frac{\exp[-r^2/(2D_{m,n})]}{(2\pi D)^{3/2}} \approx A D_{m,n}^{-3/2} \tag{15}$$

with a the maximal contact distance and A a numerical factor.

Typically, for each parameter set, we run the integration algorithm starting from 4 different initial conditions (Fig. S3): 3 from the "monophasic" regions (coil, globule and microphase) and one that mimics the experimental HiC-maps.
The experimental-like matrices for a given epigenetic pattern were obtained from the experimental map by: (1) constructing an "average" map $\bar{P}$ by assigning to every couple of monomers the average contact frequency between the epigenomic domains where they respectively belong to; (2) computing the corresponding matrix D using $D_{m,n} = A' P_{m,n}^{-2/3}$, A' being chosen such that typical intra-domain distances were of order 1.

2.2.4 Equivalence with the self-consistent method of Timoshenko, Kuznetsov and Dawson

The approach developped by Timoshenko et al. {Timoshenko et al., Phys. Rev. E, **57**: 6801 (1998); Timoshenko et al., J. Chem. Phys., **117**: 9050 (2002) } consists in approximating at each time point Eq.3 by a set of Langevin equations with a general quadratic potential

$$\xi \frac{d\mathbf{X}_n}{dt} = -\sum_m V_{n,m}(t)\mathbf{X}_m + \boldsymbol{\eta}_n(t) \tag{16}$$

From this set of equations, one can derive easily the dynamics of matrix C

$$\xi \frac{dC}{dt} = -(VC + CV^\dagger) + N \tag{17}$$

with $N = 2k_BT/\xi\mathbf{I}$. The self-consistency is given by solving

$$\sum_k V_{m,k}C_{n,k} + V_{n,k}C_{m,k} = \langle X_m \frac{\partial H}{\partial X_n} + X_n \frac{\partial H}{\partial X_m} \rangle \tag{18}$$

Timoshenko et al find that, for $n \neq m$,

$$V_{n,m} = -\frac{2}{3}\frac{\partial \langle H \rangle}{\partial D_{n,m}} \tag{19}$$

with

$$\langle H\rangle = \sum_{n<m} \sqrt{\frac{2}{\pi D_{n,m}^3}} \int_0^\infty dr r^2 U_{n,m}(r) \exp[-r^2/(2D_{n,m})] \tag{20}$$

It is easy to verify that $-V_{n,m} = \langle J_{n,m}\rangle$. This means that Eqs.10 and 17 are identical and proove that the two approaches are equivalent.

2.3 Numerical simulations

In addition to the Gaussian self-consistent approximation, we also perform full numerical simulations of the model for some parameter values.

The dynamics of the chain is described by the general equations

$$m\frac{d^2\mathbf{X}_n}{dt^2} = -\frac{\partial H}{\partial \mathbf{X}_n} - \xi\frac{d\mathbf{X}_n}{dt} + \boldsymbol{\eta}_n(t) \tag{21}$$

with m the mass of one bead. The two last terms of Eq.21 represents the coupling with the heat bath. Note that Eq.3 is derived from Eq.21 by neglecting the inertia of the system.

We choose k_BT as the unit of energy, l as the unit of length, m as the unit of mass and $\sqrt{ml^2/(k_BT)}$ as the unit of time. Simulation of trajectories are performed using the standard velocity-Verlet algorithm coupled to the Andersen thermostat (Frenkel and Smit, *Understanding molecular simulations: from algorithm to applications*, Academic Press). The velocity-Verlet algorithm allows to integrate the first - thermostat independent - part of Eq.21 ($md^2\mathbf{X}_n/dt^2 = -\partial H/\partial\mathbf{X}_n$). The Andersen thermostat accounts for the coupling with the heat bath ($-\xi d\mathbf{X}_n/dt + \boldsymbol{\eta}_n(t)$) : at a given frequency, stochastic collisions are applied to every particles of the system re-sampling the velocities among the canonical ensemble. High frequencies are associated to strong friction coefficients. In Figs.2 and 3 of the main text and in Fig. S2 of the Supplemental Material, we perform our simulation with a frequency of 5.
